# Preliminary orthodontic insights into facial soft tissue thickness measurements using semi-automated cephalometric analysis in a Cambodian cohort

**DOI:** 10.3389/fdmed.2025.1714360

**Published:** 2025-12-17

**Authors:** Eang Sonita, Meng Sally, Chhay Kimheng, Anand Marya, Prasad Nalabothu, Lao Ryna, Hiroyasu Kanetaka, Arofi Kurniawan, Abedelmalek Kalefh Tabnjh, Siddharthan Selvaraj

**Affiliations:** 1 Faculty of Dentistry, University of Puthisastra, Phnom Penh, Cambodia; 2 Department of Forensic Odontology, Faculty of Dental Medicine Universitas Airlangga, Surabaya, Indonesia; 3 Division of Interdisciplinary Co-Creation (ICC-Division), Liaison Center for Innovative Dentistry Graduate School of Dentistry, Tohoku University, Sendai, Japan; 4 Department of Pediatrics Oral Health and Orthodontics, University Center for Dental Medicine UZB, Basel, Switzerland; 5 Division of Orthodontics and Dentofacial Orthopedics, Tohoku University Graduate School of Dentistry, Sendai, Japan; 6 Department of Forensic Odontology, Faculty of Dental Medicine, Universitas Airlangga, Surabaya, Indonesia; 7 Department of Cariology, Institute of Odontology, The Sahlgrenska Academy, University of Gothenburg, Gothenburg, Sweden; 8 Department of Applied Dental Sciences, Faculty of Applied Medical Sciences, Jordan University of Science and Technology, Irbid, Jordan; 9 Dental Research Unit, Center for Global Health Research, Saveetha Medical College and Hospital, Saveetha Institute of Medical and Technical Sciences, Saveetha University, Chennai, India; 10 Department of Dental Research Cell, Dr.D.Y. Patil Dental College & Hospital, Dr.D.Y. Patil Vidyapeeth, Pune, India

**Keywords:** facial soft tissue thickness, lateral cephalogram, skeletal classification, semi-automated tracing, soft tissue profiling

## Abstract

**Background:**

The facial soft tissue thickness [FSTT] is a prominent factor in orthodontic diagnoses and treatment planning. The variance in FSTT measurements then gives orthodontists a better understanding of how to shape their treatment (based on classification of skeletal deformity and sex). This study aimed to assess the FSTT differences in a population of Cambodian adults aged 18–25 (in skeletal classes I, II, and III) and between genders.

**Method:**

A retrospective audit of 300 lateral cephalometric radiographs was undertaken through UP Dental Hospital. The sample was stratified equally by sex and skeletal class (*n* = 100 per class; 50 males, 50 females) based on Steiner analysis SNA, SNB and ANB. Linear FSTT measurements were carried out at 11 points using the software Webceph.

**Results:**

Significant FSTT differences were measured at the various cephalometric landmarks; in males, significant difference is noted at Rhinion, Subnasale, Labrale superius, and Stomion. In females, significant differences exist at Subnasale, Labrale superius, Stomion, and Labrale inferius. Males had a thicker soft tissue than females when evaluated collectively among the 11 points and skeletal Classes.

**Conclusion:**

FSTT helps in determining individualized treatment plans. Of the modalities of imaging that are available, lateral cephalometric radiography still represents the gold standard for determining facial soft tissue. This study provides a baseline reference for orthodontists, maxillofacial surgeons, and dental surgeons in Cambodia for treatment planning. Knowledge of the differences in FSTT could contribute to more customized treatment plans.

## Introduction

The facial soft tissue thickness (FSTT) measurement is an important key feature to determine treatment planning for certain procedures. It varies between one individual to another depending on race, gender, ethnicity, and age. There has been a lot of studies on FSTT these past years and it follows the same principles as FSTT are measured by various tools or methodology (1). Apparently, knowledge in this field will help practitioners define one's treatment planning according to their classifications. Recent studies have shown that an accurate treatment plan in this topic results in better postoperative results. Hence, some even reported to have better surgical planning (2, 3). Nearly all research adheres to the same guidelines, which Welcker (4) first proposed in 1883, on the measurement of tissue thicknesses.

FSTT variations help in customized treatment planning for each individual (1). This study will provide an opinion on FSTT of Cambodian populations between the ages 18–25 years old between both genders. It has been reported that FSTT plays an important role in defining facial feature of an individual (4).

The FSTT varies from each person and plays a major role in management of many treatment options related to orthodontics as the changes in a patient's soft tissue profile affects the success of orthodontic treatment. Assessment of FSTT in an individual is an important factor that clinicians need to keep in mind in addition to accounting for the skeletal malocclusion and soft tissue dimensions while planning the treatment protocol (5). Over the past few years, the hard tissue paradigm has given way to the soft tissue paradigm with more emphasis being laid on the surrounding soft tissues.

Using lateral cephalography the FSTT can be measured semi automatedly after plotting 11 points including Glabella (G), Nasion (N), Rhinion (Rhi), Subnasale (Sn), Labrale superius (Ls), Stomion (Sto), Labrale inferius (Li), Labriomentale (Labm), Pogonion (Pog), Gnathion (Gn) and Menton (Me).

There have been studies before related to variation of facial soft tissues in different populations such as Nepal, Korea, Turkey, India, Italy, Pakistan and Brazil (6–12). The study by Hasan Kamaka and Mevlut Celikoglub, showed that the thickness of facial soft tissue at the Labrale superius in class III patients was significantly different compared to class I and II patients in both males and females, and that men were found to have greater tissue thickness than women in several points including Labrale superius, stomion, and Labrale inferius (13). A study on Nepalese adolescents with class I, II, III profiles revealed that there were significant differences involving soft tissues thickness at 11 points (14). In another study in Serbia, men with class I and class II division 2 orthodontic malocclusions had thicker facial soft tissues in the mentolabial sulcus and chin areas compared with female patients, while female patients with Class II Division 1 malocclusions had thicker facial soft tissues in the mentolabial sulcus and chin compared with men (15). The FSTT of men and women with a class III skeletal jaw relationship did not differ significantly (5).

Studies also suggest that there is a wide variety of FSTT profiles in different populations. However, until now the FSTT pattern within the Cambodian population is still unknown. Therefore, a very important factor to consider during the orthodontic treatment planning process is lacking. The aim of the present study was to measure and compare facial soft tissue thickness across different Orthodontic Malocclusions in the male and female population in Cambodia.

In this study, the facial profile was evaluated by measuring facial soft tissue thickness (FSTT) at multiple anatomical landmarks across different skeletal classes, as determined by the ANB angle. All cephalometric radiographs were analyzed using a semi-automated method with WebCeph software (Assemble Circle Corp., Republic of Korea). Comparative analyses were also performed between male and female participants. Furthermore, this investigation provides baseline FSTT data for young adults in Cambodia, which may serve as a valuable reference for orthodontic diagnostics, treatment planning, and presurgical assessment, thereby supporting clinicians and surgeons in optimizing patient care.

The present findings provide a valuable reference for orthodontists, dentists, maxillofacial surgeons, and dental researchers. To the best of our knowledge, this is the first study to investigate facial soft tissue thickness (FSTT) in a Cambodian population. The results can be compared with data from other countries and ethnic groups to identify population-specific variations in FSTT. Such information is of particular importance for clinicians during diagnosis and treatment planning, especially in assessing regions prone to change during and after orthodontic treatment, such as the nasolabial area and lip profiles.

## Materials and methods

This study employs quantitative methodology and can be regarded as a pilot study. Convenience sampling was utilized for this study. The study design is appropriate as we aim to not only establish baselines values for the Cambodian population but also compare Cambodian data with data from similar studies in other populations. The total study population included in this study was 300 samples. The study was a retrospective audit of lateral cephalograms previously taken in the University of Puthisastra Dental Hospital for the purpose of the routine orthodontic assessment and treatment (thus avoiding unnecessary exposure of new patients to radiation). Ethics approval was obtained from the University of Puthisastra Research Committee (UPRC).

### Data collection

A convenience sample of the tracings of 300 lateral cephalograms were randomly selected and then divided into 3 broad categories of 100 samples each (50 male and 50 female patients) – Skeletal Class I, II and III based on Steiner skeletal analysis (sagittal) on three main values: SNA, SNB and ANB angles. Once these radiographs were divided into three groups then semi-automated tracing of the lateral cephalogram was done using Webceph application. The only demographic information recorded the age and the gender of the participants.

First examiner indicated the initial landmarks and conducted all the manual adjustments to eliminate inter-examiner bias. Second examiner reviewed 20% of the randomly selected tracings for reliability purposes to assess intra- and inter-observer reliability.

**Figure 1:**
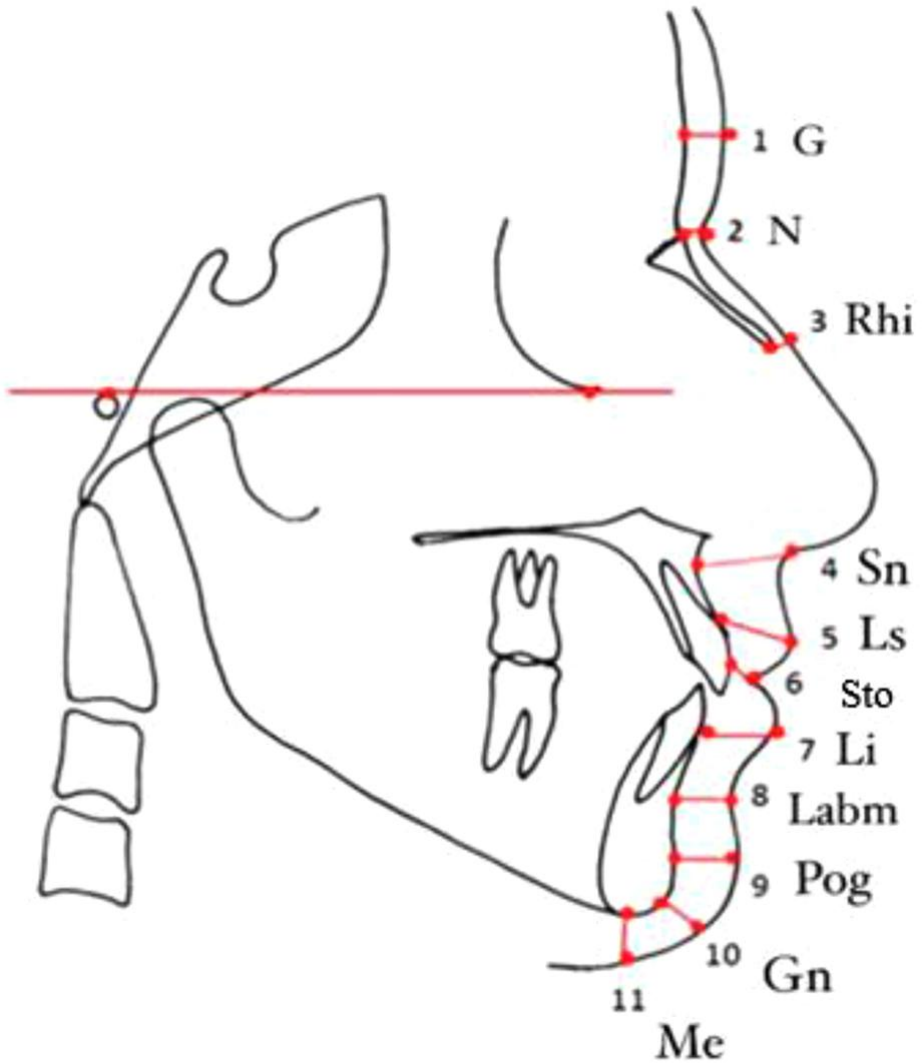
Landmarks and reference points.

The radiographs were measured semi-automatedly across 11 points including Glabella (G), Nasion (N), Rhinion (Rhi), Subnasale (Sn), Labrale superius (Ls), Stomion (Sto), Labrale inferius (Li), Labriomentale (Labm), Pogonion (Pog), Gnathion (Gn) and Menton (Me). Landmarks and reference points are illustrated in Figure 1.

Explicit cut-off values in our study are as follows:

Class I: ANB 0°–4°
Class II: ANB >4°
Class III: ANB <0°

SNA and SNB were used descriptively to characterize maxillary and mandibular positions, but ANB angle served as the primary classifier.

### Inclusion and exclusion criteria

Male and female adults aged between 18 and 25 years were included in this study. Lateral cephalogram not of acceptable quality, e.g., Had distortions, overlapping, unclear and individuals who had received orthodontic treatment previously were excluded from this study.

### Data analysis

Data collected in this study was analyzed using SPSS statistical software. Frequency tables were generated. Subgroup analysis between males and females and classes of skeletal malocclusions were carried out. Statistical tests included Mann–Whitney *U* for two-group comparisons and Kruskal–Wallis for multiple-group comparisons. The data was analyzed based on gender related changes as well as the patient's skeletal classification and the mean age of each sample category.

## Results

A total of 300 participants, 150 (50%) male and 150 (50%) female participants were included in this study. There were equal number (100 each) of participants in each class (I, II and III).

The mean difference of facial soft tissue thickness between male and female participants are presented in Table 1. The results reveal a significant mean difference between male and female participants in Nasion (*P* < 0.001), Rhinion (*P* = 0.028), Subnasale (*P* < 0.001), Labrale superius (*P* < 0.001), Stomion (*P* = 0.016), Labrale inferius (*P* = 0.002), and Menton (*P* = 0.031). The mean FSTT in males was significantly greater than in females at Nasion (6.54 vs. 5.87), Rhinion (2.79 vs. 2.56), Subnasale (14.62 vs. 13.58), Labrale superius (14.58 vs. 13.40), Stomion (6.23 vs. 5.30), Labrale inferius (14.94 vs. 14.09), and Menton (8.06 vs. 7.36).

**Table 1:** Mean difference of facial soft tissue thickness between male and female participants.

Variable	Male Mean (SD)	Female Mean (SD)	*Z*	*p*-value
Glabella (G)	5.30 (1.08)	5.22 (0.93)	−0.047	0.962
Nasion (N)	6.54 (1.38)	5.87 (1.02)	−4.526	**<0.001**
Rhinion (Rhi)	2.79 (0.92)	2.56 (0.68)	−2.201	**0.028**
Subnasale (Sn)	14.62 (2.89)	13.58 (2.49)	−3.240	**0.001**
Labrale superius (Ls)	14.58 (2.67)	13.40 (2.28)	−3.863	**<0.001**
Stomion (Sto)	6.23 (3.02)	5.30 (2.32)	−2.401	**0.016**
Labrale inferius (Li)	14.94 (2.20)	14.09 (1.83)	−3.125	**0.002**
Labiomentale (Labm)	12.45 (2.02)	12.11 (1.66)	−1.439	0.150
Pogonion (Pog)	11.65 (2.45)	11.46 (2.17)	−0.539	0.590
Gnathion (Gn)	8.73 (2.88)	8.45 (2.29)	−0.225	0.799
Menton (Me)	8.06 (2.52)	7.36 (1.95)	−2.162	**0.031**

Statistically significant values are bolded.

The mean difference of facial soft tissue thickness between classes are illustrated in Table 2. The results reveal a significant mean difference between classes in Glabella (*P* < 0.001), Rhinion (*P* < 0.001), Subnasale (*P* < 0.001), Labrale superius (*P* < 0.001), Stomion (*P* < 0.001), Labrale inferius (*P* < 0.001), Labiomentale (*P* < 0.001), Pogonion (*P* = 0.005), Gnathion (*P* = 0.019), and Menton (*P* = 0.019). The results show that Glabella (5.66), Rhinion (2.89), Pogonion (11.94), and Gnathion (9.17) have a higher mean in class I. In class II, Labrale inferius (15.32) and Labiomentale (12.71) have a higher mean. In class III, Subnasale (15.26), Labrale superius (15.21), and Stomion (7.65) have a higher mean.

**Table 2:** Mean difference of facial soft tissue thickness between class types.

Variable	Class I Mean (SD)	Class II Mean (SD)	Class III Mean (SD)	*Z*	*p*-value	Pairwise
Glabella (G)	5.66 (1.12)	5.12 (0.89)	4.99 (0.86)	23.593	**<0.001**	*Class I vs. Class II (p* *<* *0.001*) *Class I vs. Class III (p* *<* *0.001)* *Class II vs. Class III (p* *=* *0.400)*
Nasion (N)	6.23 (1.42)	6.17 (1.18)	6.22 (1.18)	0.118	0.943	–
Rhinion (Rhi)	2.89 (0.90)	2.46 (0.65)	2.69 (0.84)	21.155	**<0.001**	*Class I vs. Class II (p* *<* *0.001)* *Class I vs. Class III (p* *=* *0.011)* *Class II vs. Class III (p* *=* *0.041)*
Subnasale (Sn)	14.01 (2.36)	13.03 (2.30)	15.26 (3.06)	35.094	**<0.001**	*Class I vs. Class II (p* *=* *0.007)* *Class I vs. Class III (p* *<* *0.001)* *Class II vs. Class III (p* *<* *0.001)*
Labrale superius (Ls)	13.64 (2.24)	13.13 (2.30)	15.21 (2.61)	39.672	**<0.001**	*Class I vs. Class II (p* *=* *0.150)* *Class I vs. Class III (p* *<* *0.001)* *Class II vs. Class III (p* *<* *0.001)*
Stomion (Sto)	5.86 (2.07)	3.78 (1.36)	7.65 (2.97)	110.766	**<0.001**	*Class I vs. Class II (p* *<* *0.001)* *Class I vs. Class III (p* *<* *0.001)* *Class II vs. Class III (p* *<* *0.001)*
Labrale inferius (Li)	14.81 (2.05)	15.32 (1.97)	13.43 (1.69)	52.326	**<0.001**	*Class I vs. Class II (p* *=* *0.014)* *Class I vs. Class III (p* *<* *0.001)* *Class II vs. Class III (p* *<* *0.001)*
Labiomentale (Labm)	12.48 (1.86)	12.71 (1.83)	11.65 (1.71)	20.708	**<0.001**	*Class I vs. Class II (p* *=* *0.274)* *Class I vs. Class III (p* *=* *0.001)* *Class II vs. Class III (p* *<* *0.001)*
Pogonion (Pog)	11.94 (2.36)	11.79 (2.46)	10.93 (1.99)	10.777	**0.005**	*Class I vs. Class II (p* *=* *0.577)* *Class I vs. Class III (p* *=* *0.002)* *Class II vs. Class III (p* *=* *0.012)*
Gnathion (Gn)	9.17 (2.69)	8.39 (2.69)	8.22 (2.33)	7.918	**0.019**	*Class I vs. Class II (p* *=* *0.030)* *Class I vs. Class III (p* *=* *0.008)* *Class II vs. Class III (p* *=* *0.639)*
Menton (Me)	7.87 (2.24)	8.39 (2.28)	6.87 (2.06)	29.602	**<0.001**	*Class I vs. Class II (p* *=* *0.106)* *Class I vs. Class III (p* *<* *0.001)* *Class II vs. Class III (p* *<* *0.001)*

Statistically significant values are bolded.

## Discussion

Facial Soft Tissue adaptation to orthodontic or orthognathic treatment does not precisely correlate one-to-one to either tooth or skeletal movement (16–18). A study by Han and his colleagues in the year 2022 among adult patients illustrated that variations in soft-tissue adaptation occurred with moderate change of facial appearance that was only partially related to dental or skeletal displacement (19). Pre-treatment facial profiles as well as treatment mechanics vary the magnitude and pattern of soft-tissue change (13, 20).

Soft-tissue response to treatment is inherently multifactorial. Lip thickness, muscle function, soft-tissue elasticity, and the skeletal structure all contribute to the extent to which the facial appearance will be altered after orthodontic treatment (19, 21, 22). Also, advances in three-dimensional imaging and volumetric evaluation have made it possible to more accurately assess soft-tissue adaptation and demonstrate that soft-tissue displacement is generally less than hard-tissue adaptation (19, 23–25).

FST adaptation to orthodontic or orthognathic treatment does not precisely correlate one-to-one to either tooth or skeletal movement. Soft tissue reaction is multifactorial and influenced by many variables including the both direction and amount of the dental and skeletal movement, the thickness and elastic properties of the initial tissue, muscle tone, lip posture and morphology of the skeletal structure. Therefore, soft-tissue thickness alone is insufficient to reliably predict aesthetic outcome (16–18). Recently, variability of soft-tissue adaptation has been highlighted. A study in adult patients illustrated that variations in soft-tissue adaptation occurred with moderate change of facial appearance that was only partially related to dental or skeletal displacement (19). Pre-treatment facial profiles as well as treatment mechanics vary the magnitude and pattern of soft-tissue change (13, 20). Systematic reviews further suggest that extraction vs. non-extraction protocols significantly influence lip retraction and the nasolabial angle, however, certainty of evidence remains low with high variation among individuals (16, 17). Soft-tissue response to treatment is inherently multifactorial. Lip thickness, muscle function, soft-tissue elasticity, and the skeletal structure all contribute to the extent to which the facial appearance will be altered after orthodontic treatment (19, 21, 22). Also, advances in three-dimensional imaging and volumetric evaluation have made it possible to more accurately assess soft-tissue adaptation and demonstrate that soft-tissue displacement is generally less than hard-tissue adaptation.

The paradigm of dentistry is changing from hard-tissue-based treatment to soft-tissue-based treatment as the desire for face aesthetics rises. Facial analysis requires considering the soft tissue as well because the features of the face cannot be anticipated based only on the hard tissue (26).

The 2D imaging methods have the advantages of quick acquisition times, low costs, and archival possibilities. Limitations include exposure to ionizing radiation, parallax, head orientation and measurement inaccuracy caused by magnification. Three-dimensional (3D) digital imaging is now a common practice for craniofacial researchers and doctors thanks to recent technology breakthroughs. Techniques like cone beam computed tomography (CBCT) (6), surface laser scanning (7, 8) and stereophotogrammetry (9–11) became available to describe and compare 3D facial surfaces, create a diagnosis or virtual treatment planning as well as to evaluate growth and treatment outcomes. These methods allow images to be archived and avoid measurement errors that occur with 2D representations of 3D surfaces. CBCT however, is not an ideal technique for surface measurement because of poor resolution of facial contours, high cost and exposure to ionizing radiation (12). For the comparatively low cost, laser surface scanning can be a dependable and accurate method of identifying landmarks on the craniofacial surface. Limitations include the risk for eye injury and slow image capturing (up to 20 s) (27).

However, in 2022, Kılınç (28) provided an example of computerized cephalometric tracing. There are a lot of tools on the market after that. Regretfully, these instruments are exceedingly costly and cannot be modified for application in the examination of the cephalometric research. Consequently, it is imperative to find a solution for the soft tissue profile analysis examination. Other technologies also have the drawback of not automatically providing linear and angular measurements; instead, measurements for each study subject must be taken using a ruler, which is a laborious and time-consuming task.

### Soft tissue analysis between male and female on 3 skeletal classes at 11 different points

In this study, we measured facial soft tissue thickness of adult male and female subjects with three different sagittal skeletal malocclusions at 11 points i.e., Glabella (G), Nasion (N), Rhinion (Rhi), Subnasale (Sn), Labrale superius (Ls), Stomion (Sto), Labrale inferius (Li), Labiomentale (Labm), Pogonion (Pog), Gnathion (Gn) and Menton (Me). When facial soft tissue thickness was compared among three sagittal skeletal classes, we found significant differences at points Rhinion, Subnasale, Labrale superius and Stomion in males and at Subnasale, Labrale superius, Stomion and Labrale inferius in females. As a result, FSTT of males are thicker than females in all 11 points across 3 different skeletal classes.

### Importance of facial soft tissue thickness data for different type of treatment plan

Gender influences the thickness of soft tissue in various facial locations. For every gender, orthodontists ought to choose a distinct therapeutic approach (15). When planning a treatment procedure, plastic surgeons and orthodontists consider the thickness of the soft tissues in the face. Even though the skull's bony structure can provide certain details about the facial features of an individual, it cannot provide sufficient information on its own. Soft tissue and skeletal structure work together to create harmony and balance in the face. Even though some people may not always offer precise soft tissue data, FSTT is nevertheless crucial for treatment planning (29). Retracting, maintaining, or protracting the upper and/or lower lip; increasing, maintaining, or decreasing vermilion display (lip thickness); reducing lip strain, mentalis muscle strain, and interlabial gap or maintaining lip competence; increasing, maintaining, or decreasing nasolabial angle; increasing, maintaining, or decreasing mentolabial angle; increasing or maintaining cervicomental angle; improving facial asymmetry; increasing, maintaining, or decreasing width of the alar base; and increasing, maintaining, or decreasing the vertical and/or antero-posterior projection of the soft tissue chin are some of the soft tissue objectives that must be taken into account during the treatment planning process. The literature on soft tissue changes with growth, soft tissue changes with treatment (effects of extraction and non-extraction therapy, headgear and functional appliances, protraction facemask, orthognathic surgery), and the smile is reviewed in order to formulate these treatment objectives for each patient individually (30).

### Comparison between male and female facial soft tissue thickness

It has been revealed in multiple cephalometric researches that males have much larger facial soft-tissue thickness (FSTT) compared to females at nearly every midline landmark, regardless of skeletal class (31, 32). For example, males have greater FSTT than females at the nasion, subnasale, labrale superius, stomion, labrale inferius, and menton (33). This pattern has produced across Class I, II, and III skeletal patterns and suggests that sex is an important factor in soft-tissue composition (32, 34). These differences may arise from skeletal size, soft-tissue composition, hormonal influences, or any combination of these factors (34, 35). These results emphasize the importance of considering gender in orthodontic diagnosis and treatment planning, facial profile predictions, and forensic purposes (31, 35).

We measured 300 samples of equally distributed by gender, across three different skeletal classes during the course of the study. In Nasion, Rhinion, Subnasale, Labrale superius, Stomion, Labrale inferius and Menton, the mean differences between males and females reveal a significant mean difference, according to our results. The male possesses a thicker FSTT than female in all 11 points across the face.

Comparative studies on Asian populations with similar craniofacial features have revealed soft tissue thickness variations that are almost identical to the present research. Our findings - males have significantly higher soft tissue thickness at Rhinion, Subnasale, Labrale superius, and Stomion - are corroborated by research on Chinese and Korean adults where the mid-facial and perioral soft tissues are reported to be consistently thicker in males than in females (36–38).

Moreover, research on Japanese, Thai, and Malaysian populations show that male FSTT is significantly higher at subnasal and labial landmarks, thus reflecting the sexual dimorphism in our sample, which has been confirmed by these studies as well (39–41). In addition, studies on South Asian and the entire Southeast Asian populations reveal that females have relatively thinner soft tissues at Labrale superius, Stomion, and Labrale inferius which is in agreement with the differences observed in our data (42, 43).

These patterns, in essence, point to a strong regional agreement of facial soft-tissue profiles for the different Asian populations and the extension of our results is very plausible in forensic, orthodontic, and anthropological contexts.

## Conclusion

Facial soft tissue thickness (FSTT) helps in determining the most appropriate and individualized treatment plan. Accurate assessment of FSTT assists orthodontists in optimizing treatment planning to achieve the best functional and esthetic outcomes for patients. In this study, lateral cephalometric radiographs were used to evaluate FSTT across 11 anatomical landmarks. The research results showed that men always had more FSTT than women. In fact, significant mean differences at the Nasion, Rhinion, Subnasale, Labrale superius, Stomion, Labrale inferius, and Menton were specifically pointed out. These findings emphasize the necessity of using FSTT measurements in orthodontic diagnostics and treatment planning.

## Data Availability

The original contributions presented in the study are included in the article/Supplementary Material, further inquiries can be directed to the corresponding authors.
